# Disparities in maternal mortality

**DOI:** 10.1017/cts.2023.520

**Published:** 2023-04-20

**Authors:** Phuong Tran, Barbara Jreij, Farideh Sistani, Fadia T. Shaya

**Affiliations:** 1 Department of Practice, Sciences and Health Outcomes Research, University of Maryland School of Pharmacy, Baltimore, MD, USA; 2 Faculty of Medicine and Medical Sciences, University of Balamand, Balamand, Lebanon

**Keywords:** Maternal mortality, racial disparities, age disparities, miscarriage, public health

## Abstract

Maternal mortality rates in the USA remain high, with persistent racial and socioeconomic disparities. We identified 207,016 hospital admissions for pregnant women in Maryland, from 2017 to 2019. Logistic regression was used to identity factors associated with maternal death. The health outcome for black women was more prone to give rise to maternal mortality than for white women. Our study revealed numerous racial and age discrepancies in gestational health outcomes, which opioid use disorder exacerbated. Our findings elaborate on the importance of identifying the drivers of adverse pregnancy outcomes, to help inform policy, and resource allocations.

## Introduction

Despite the increased spending on prenatal care and efforts to improve reproductive health care access for women, maternal mortality rates in the USA remain high, with persistent racial and socioeconomic disparities [1]. Several studies have provided data demonstrating the risks women of color undergo because of lesser healthcare access and quality. The USA has a significantly higher maternal mortality rate than other comparable countries. Approximately 700 women die in the USA each year because of pregnancy or its complications [1]. According to the Centers for Disease Control and Prevention (CDC), cardiomyopathy, thrombotic pulmonary embolism, and hypertensive disorders of pregnancy were among the key factors that contributed to a significantly higher proportion of pregnancy-related deaths among black women compared to white women [1].

Furthermore, it should also be noted that women of color have been reported to experience inequalities based on their age and level of education. As for the educational level, it has been reported that the pregnancy-related mortality rate for black women with a completed college education or higher is 1.6 times higher than the rate for white women with less than a high school diploma [1]. Therefore, these acts of discrimination in the healthcare sector are present and persist regardless of one’s educational level and socioeconomic status. Most of these deaths are preventable, which has been mentioned on several accounts. However, due to the racism, black women are more likely to die from complications of pregnancy than white women [2]. For instance, the risk of cardiomyopathy, leading cause of late maternal death, in black women is six times higher than in white women [3]. During a study carried out in 2007–2016, pregnancy-related deaths for all US women were reported to be 16.7 deaths per 100,000 live births. When taking a closer look at these numbers among different ethnic groups, the difference in the death rate among black women becomes noticeable. The deaths among white women were reported to be 12.7 deaths per 100,000 live births, while those among black women reached 40.8 deaths per 100,000 live births [1].

In addition, women with opioid use disorder (OUD) suffer from increased pregnancy complication risks. In fact, the number of pregnant women with OUD has increased four times in the past decade [4]. This can be explained by the fact that there has been a decrease in the administration of the OUD treatment in the general population because of shortages, insufficient insurance funds, stigma, and a general miscomprehension of the disorder [5,6]. However, when considering smaller and more specific racial/ethnic populations such as the black communities, it has been observed that there is less access to the OUD treatment, buprenorphine, than in white non-Hispanic, non-Black people, along with a less timely administration of the medication [7,8]. In parallel, OUD complications have not spared pregnant women, thus possibly contributing to this high maternal mortality rates [1]. These trends are especially noted in Maryland [4], thus, it is important to identify the drivers of adverse pregnancy outcomes to help inform policy and resource allocations.

This study aimed to identify and assess determinants of maternal mortality or miscarriage using inpatient discharge data from all Maryland hospitals between 2017 and 2019.

## Methods

We extracted data using a patient-level dataset available through the Hospital Data and Reporting system in Maryland [9]. The dataset contains discharge medical record abstract and billing data from all acute care hospitals and licensed specialty hospitals in the state. We identified 207,016 hospital admissions in Maryland for pregnant women ages 14–45 years from January 2017, through December 2019. We categorized our predictor variables under three main categories: comorbidities, clinical factors, and demographics. The primary outcome was maternal mortality or miscarriage. All diagnoses were identified through ICD-10 codes. To identify pregnant patients, we used ICD-10 codes Z34 and Z33.1. For comorbidities, we examined OUD (ICD-10 codes: F11.1 and F11.2), high-risk pregnancy (ICD-10 code: O09), preexisting hypertension (ICD-10 codes: O10–O16), and diabetes (O24). For clinical factors, we looked at the nature of admission such as delivery, scheduled, emergency and urgent, other type of admissions, and major hospital service to which patients were assigned, including psychiatric and other types of services. We examined age, race, patient residency in Maryland, and primary payer for demographic variables. Due to the very low prevalence of maternal mortality, we used a composite outcome of maternal mortality and miscarriage.

We performed a bivariate analysis on all predictor variables using logistic regression to explore any association between each characteristic and maternal mortality or miscarriage. Variables that showed a statistically significant association with the outcome, in addition to age as an important confounder, were selected as candidate variables for the multivariate analysis to detect risk factors of maternal mortality or miscarriage after adjusting for other covariates. P < 0.05 was considered to be statistically significant, and all statistical analyses were performed with SAS version 9.4 (SAS Institute, Cary, NC).

## Results

Between 2017 and 2019, out of 207,016 hospital admissions, there were 66,311 (32%) hospital admissions for black women and 3014 (1.46%) hospital admissions for women with OUD. About 16% of 207,016 hospital admissions were for women aged 35 years or older and 4% for women 14–19 years old (Table 1). There were 587 hospital admissions where pregnant women either died or had a miscarriage (28 per 10,000 hospital admissions).

**Table 1.** Hospital admissions by age group, race and opioid use disorder status

Demographic	Hospital Admissions n (%) (N = 207016)
Age	
14–19 years old	8443 (4.1)
20–35 years old	164,309 (79.4)
35–45 years old	34,264 (17.5)
Race	
White	91,381 (44.1)
Black	66,311 (32)
Asian	12,527 (6.1)
Other	36,797 (17.8)
Opioid Use Disorder (OUD) Status	
No	204,002 (98.5)
Yes	3014 (1.5)

The findings of the multivariate-adjusted model indicated that among comorbidities examined in this study, diabetes (OR = 0.42, 95%CI: 0.27–0.65), preexisting hypertension (OR = 0.30, 95%CI: 0.22–0.41), and OUD (OR = 2.61, 95%CI: 1.71–3.99) were significantly associated with maternal mortality or miscarriage. High-risk pregnancy was not associated with adverse maternal health outcomes (p = 0.23).

Patients with Medicaid insurance (OR = 0.82, 95%CI: 0.68–1) were at a higher risk of maternal mortality or miscarriage than those with commercial insurance. Moreover, we found that after controlling for residency status, nature of hospital admissions, major hospital service assigned, and comorbidities (high-risk pregnancy, hypertension, diabetes, and OUD) there were still substantial racial and age disparities in hospital admissions, with adverse pregnancy-related outcomes. Hospital admissions for Black women were more likely to result in maternal mortality and miscarriage (OR = 2.16, 95%CI: 1.76–2.65) than for White women. Likewise, advanced maternal age (35–45 years old) (OR = 1.79, 95%CI: 1.45–2.20) was associated with adverse outcomes (Table 2).

**Table 2.** Factors associated with maternal mortality and miscarriages among hospital admissions of pregnant women between 2017 and 2019

Variables	Number (%) of visits with maternal mortality or miscarriage	Univariate model		Multivariable model	
		Crude odds ratio (OR) (95%CI)	p-value	Adjusted odds ratio (OR) (95%CI)	p-value
*Comorbidities*					
Opioid use disorder					
No	562 (0.28%)	1		1	
Yes	25 (0.83%)	**3.03 (2.03–4.53)**	**<0.0001**	**2.61 (1.71–3.99)**	**<0.0001**
Supervision of high-risk pregnancy					
No	556 (0.28%)	1			
Yes	31 (0.29%)	1.03 (0.72–1.48)	0.872	0.80 (0.55–1.16)	0.2331
Pre-existing hypertension complicating pregnancy, childbirth, and the puerperium					
No	545 (0.31%)	1		1	
Yes	42(0.12%)	**0.39 (0.29–0.54)**	**<0.0001**	**0.30 (0.22–0.41)**	**<0.0001**
Diabetes mellitus in pregnancy, childbirth, and the puerperium					
No	567 (0.30%)	1		1	
Yes	20 (0.10%)	**0.34 (0.22–0.53)**	**<0.0001**	**0.42 (0.27-0.65)**	**0.0001**
*Clinical factors*					
Nature of admission					
Delivery	91(0.07%)	1		1	
Scheduled	32 (0.10%)	**1.51 (1.01- 2.27)**	**<0.0001**	**1.54 (1.03–2.30)**	**0.0368**
Emergency and urgent	457 (1.33%)	**10.60 (16.45–25.81)**	**<0.0001**	**20.46 (16.25–25.76)**	**<0.0001**
Other	7 (0.70%)	**10.76 (4.98–23.67)**	**0.001**	**7.15 (3.06-16.67)**	**<0.0001**
Major hospital service to which the patient was assigned					
Psychiatric	15 (1.92%)	7.04 (4.20–11.84)	<0.0001	1.65 (0.93–2.95)	0.0884
Other	572 (0.28%)	1		1	
*Demographic factors*					
Patient’s age					
14–19 years old	27 (0.32%)	1.20 (0.81–1.77)	0.3639	0.85 (0.57–1.26)	0.4172
20–35 years old	439 (0.27%)	1		1	
35–45 years old	121 (0.35%)	**1.32 (1.08–1.62)**	**0.0064**	**1.79 (1.45–2.20)**	**<0.0001**
Race of the patient					
White	161 (0.18%)	1		1	
Black	314 (0.47%)	**2.70 (2.23–3.26)**	**<0.0001**	**2.16 (1.76-2.65)**	**<0.0001**
Asian	21 (0.17%)	0.95 (0.60–1.50)	0.033	1.58 (1.00–2.51)	0.0516
Other	91 (0.25%)	**1.40 (1.09–1.82)**	0.8447	**1.80 (1.37–2.35)**	**<0.0001**
Patient residency					
Maryland resident	553 (0.28%)	1		1	
Other	34 (0.49%)	1.78 (1.26–2.52)	0.001	**1.68 (1.17–2.41)**	**0.0049**
Expected primary payer					
Medicare	8 (0.66%)	**2.98 (1.47–6.05)**	**0.0542**	0.81 (0.393-1.67)	0.5688
Maryland Medicaid	323 (0.31%)	**1.39 (1.17–1.65)**	**0.0204**	**0.82 (0.68- 1.00)**	**0.0469**
Commercial	206 (0.22%)	1		1	
Self-pay and Other	50 (0.53%)	**2.40 (1.76–3.27)**	**0.0323**	**1.89 (1.37-2.61)**	**0.0001**

The bold entries in Tables 2 denote a statistically significant result.

## Discussion

This study focused on evaluating the impact of important clinical factors, comorbidities, and patient demographics on maternal mortality or miscarriage. Our analysis unveiled racial and age disparities in pregnancy health outcomes, which were exacerbated by OUD. These findings are consistent with previous studies, which found persistent racial disparities in maternal health [1,10], and increased risk of perinatal mortality and adverse obstetric outcomes with advanced maternal age [7] and OUD [11]. For instance, the results of a study by Lagisetty *et al.* demonstrated that buprenorphine treatment was concentrated among white persons and patients with private insurance [10]. Likewise, another study indicated a higher prevalence of coexisting conditions with aging, which can put pregnant women with advanced age at a two to three times higher risk of hospitalization, C-section, and other pregnancy-related complications [12]. Crear-Perry *et al.* call out racism as the root cause of inequities in maternal health outcomes [13]. A comprehensive approach to quality improvement throughout the care continuum from preconception to postpartum may help reduce disparities. One possible way would be a disparities dashboard to help monitor a system’s performance quality metrics for higher risk groups.

A multidisciplinary approach of prenatal care should be recommended for complication-prone pregnancies. In addition to systemic racism and discrimination, the difference in health insurance coverage and access to care also play a role in driving worse maternal health outcomes. Medicaid covers almost half of the births nationally [14]. Further, Medicaid expansion promotes continuity of coverage in prenatal and postpartum periods. Our results interestingly showed that in spite of Medicaid expansion, Maryland still shows disparities in pregnancy outcomes. With Medicaid expansion, rate of uninsured childbearing age women in Maryland is well below the national average (8.4%). Yet black and white women’s average uninsured in Maryland are still within one point of each other when comparing racial groups. This can result in higher chance of pregnancy-related death among black women and underscores the role of racism and discrimination in healthcare [15].

The results on the significant association between OUD and adverse outcomes call for a proactive approach of screening and referral to treatment during pregnancy. Concerted efforts in health care delivery and access policy, extending to prenatal care, should be made to address system-based drivers of racial disparities.

This study further elaborates on the effect of racial and age disparities on adverse maternal health outcomes represented by previous studies. However, our study faced some limitations that should be considered in the interpretation. First, the analysis was restricted to the Maryland hospitals, and results may not be generalizable to the nation. Second, we only included variables known or plausibly associated with maternal mortality or miscarriage. Further evaluations can include other socioeconomic factors such as income/poverty, food security, provider and pharmacy availability, and education. Third, our dataset precluded us from analyzing patients that were not admitted. Future research should study the practice population that were not admitted determining differences regarding access to care. One of the limitations of this study is that we did not adjust for some potential risk factors for adverse pregnancy outcomes such as pre-eclampsia, obesity, and mental health disorders.

## Conclusion

Improving maternal health is essential to reduce preventable maternal mortality or miscarriage and advancing overall population health. This study highlights the importance of raise in attention to disparities in maternal health. Our findings suggest increasing efforts and resources such as expanding access to coverage and care, patient education on risks associated with advanced maternal age and addressing drivers of racial disparities. Further evaluation of risk factors contributing to racial inequality is crucial to inform and implement strategies to reduce such disparities in maternal mortality and miscarriage.

## References

[1] Petersen EE , Davis NL , Goodman D , et al. Racial/ethnic disparities in pregnancy-related deaths — United States, 2007–2016. MMWR Morb Mortal Wkly Rep. 2019;68(35):762–765. doi: 10.15585/mmwr.mm6835a3. 31487273 PMC6730892

[2] Flanders-Stepans MB. Alarming racial differences in maternal mortality. J Perinat Educ. 2000;9(2):50–51. doi: 10.1624/105812400X87653. 17273206 PMC1595019

[3] PRB. Black women over three times more likely to die in pregnancy, postpartum than white women, new research finds. https://www.prb.org/resources/black-women-over-three-times-more-likely-to-die-in-pregnancy-postpartum-than-white-women-new-research-finds/. Accessed March 16, 2023.

[4] Haight SC. Opioid use disorder documented at delivery hospitalization — United States, 1999–2014. MMWR Morb Mortal Wkly Rep. 2018;67(31):845–849. doi: 10.15585/mmwr.mm6731a1. 30091969 PMC6089335

[5] National Academies of Sciences, Engineering, and Medicine, Health and Medicine Division, Board on Health Sciences Policy, Committee on Medication-Assisted Treatment for Opioid Use Disorder, Mancher M , Leshner AI. Barriers to Broader Use of Medications to Treat Opioid Use Disorder. Washington, DC: National Academies Press (US); 2019. https://www.ncbi.nlm.nih.gov/books/NBK541389/. Accessed March 17, 2023.

[6] PatientEngagementHIT. Nearly 90% of patients with opioid use disorder lack medication access. https://patientengagementhit.com/news/nearly-90-of-patients-with-opioid-use-disorder-lack-medication-access. Published August 9, 2022. Accessed March 17, 2023.

[7] Lu MC , Noursi S. Summary and conclusion: framing a new research agenda on maternal morbidities and mortality in the United States. J Womens Health. 2021;30(2):280–284. doi: 10.1089/jwh.2020.8877. PMC8020501 33216690

[8] Creanga AA , Syverson C , Seed K , Callaghan WM. Pregnancy-related mortality in the United States, 2011–2013. Obstet Gynecol. 2017;130(2):366–373. doi: 10.1097/AOG.0000000000002114. 28697109 PMC5744583

[9] The Maryland Health Services Cost Review Commission. Data. https://hscrc.maryland.gov/Pages/default.aspx. Accessed January 27, 2023.

[10] Lagisetty PA , Ross R , Bohnert A , Clay M , Maust DT. Buprenorphine treatment divide by race/ethnicity and payment. JAMA Psychiatry. 2019;76(9):979–981. doi: 10.1001/jamapsychiatry.2019.0876. 31066881 PMC6506898

[11] PubMed. Receipt of timely addiction treatment and association of early medication treatment with retention in care among youths with opioid use disorder. https://pubmed-ncbi-nlm-nih-gov.proxy-hs.researchport.umd.edu/30208470/. Accessed January 30, 2023. 10.1001/jamapediatrics.2018.2143 PMC6218311 30208470

[12] Correa-de-Araujo R , Yoon SS (Sarah). Clinical outcomes in high-risk pregnancies due to advanced maternal age. J Womens Health. 2021;30(2):160–167. doi: 10.1089/jwh.2020.8860. PMC8020515 33185505

[13] Crear-Perry J , Correa-de-Araujo R , Lewis Johnson T , McLemore MR , Neilson E , Wallace M. Social and structural determinants of health inequities in maternal health. J Womens Health. 2021;30(2):230–235. doi: 10.1089/jwh.2020.8882. PMC8020519 33181043

[14] KFF. Nov 01 URP, 2022. Racial disparities in maternal and infant health: current status and efforts to address them. https://www.kff.org/racial-equity-and-health-policy/issue-brief/racial-disparities-in-maternal-and-infant-health-current-status-and-efforts-to-address-them/. Published November 2022. Accessed January 30, 2023.

[15] Cent Child Fam. Medicaid expansion narrows maternal health coverage gaps, but racial disparities persist. https://ccf.georgetown.edu/2021/09/13/medicaid-expansion-narrows-maternal-health-coverage-gaps-but-racial-disparities-persist/. Published September 2021. Accessed March 17, 2023.

